# Host Immunity to *Malassezia* in Health and Disease

**DOI:** 10.3389/fcimb.2020.00198

**Published:** 2020-05-05

**Authors:** Florian Sparber, Fiorella Ruchti, Salomé LeibundGut-Landmann

**Affiliations:** ^1^Section of Immunology, Vetsuisse Faculty, University of Zurich, Zurich, Switzerland; ^2^Institute of Experimental Immunology, University of Zurich, Zurich, Switzerland

**Keywords:** mycobiota, commensalism, immunopathology, cutaneous immunity, *Malassezia*

## Abstract

The microbiota plays an integral role in shaping physical and functional aspects of the skin. While a healthy microbiota contributes to the maintenance of immune homeostasis, dysbiosis can result in the development of diverse skin pathologies. This dichotomous feature of the skin microbiota holds true not only for bacteria, but also for fungi that colonize the skin. As such, the yeast *Malassezia*, which is by far the most abundant component of the skin mycobiota, is associated with a variety of skin disorders, of which some can be chronic and severe and have a significant impact on the quality of life of those affected. Understanding the causative relationship between *Malassezia* and the development of such skin disorders requires in-depth knowledge of the mechanism by which the immune system interacts with and responds to the fungus. In this review, we will discuss recent advances in our understanding of the immune response to *Malassezia* and how the implicated cells and cytokine pathways prevent uncontrolled fungal growth to maintain commensalism in the mammalian skin. We also review how the antifungal response is currently thought to affect the development and severity of inflammatory disorders of the skin and at distant sites.

## Introduction

The skin, one of our body's largest organs, harbors a wide variety of microbial communities, including innocuous symbiotic organisms but also potential pathogens (Findley and Grice, 2014). Advances in our understanding of the molecular mechanisms of microbial virulence and host defense have improved the current view how dysbiosis and dysregulated immune responses against commensal microbes drive pathological conditions (Chen et al., 2018).

**Figure 1:**
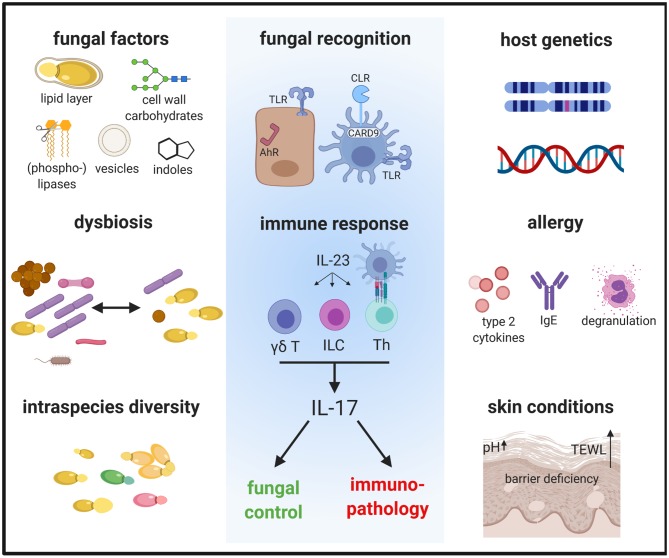
The immune response to *Malassezia* is influenced by fungal factors including cell wall constituents and secreted components, inter- and intraspecies variations and host factors such as genetics and skin or systemic predisposing conditions. These factors determine how *Malassezia* is recognized by the host and in turn how the host responds to the fungus. The antifungal response is characterized by the activation of the IL-23/IL-17 axis, which not only controls fungal growth but can also mediate immunopathology. AhR, aryl hydrocarbon receptor; CLR, C-type lectin receptor; γδ T, γδ T cells; ILC, innate lymphoid cells; TLR, Toll-like receptor; TEWL, trans-epidermal water loss; Th, T helper cells.

Fungi are increasingly being recognized as common members of the microbiota. The most prevalent fungi on the mammalian skin are those of the genus *Malassezia*, with >90% of all skin fungi belonging to this genus (Findley et al., 2013). Currently, 18 species of *Malassezia* have been identified, of which 10 were found in humans and the others in a growing number of animal hosts (Guillot and Bond, 2020). Although generally viewed as a commensal, *Malassezia* has also been associated with various dermatological conditions including mild diseases, such as dandruff and pityriasis versicolor, to more severe inflammatory diseases, such as seborrheic dermatitis and atopic dermatitis (AD) (Saunte et al., 2020). In rare cases, *Malassezia* has been reported to cause blood stream infections (Iatta et al., 2014). In dogs, overgrowth of *Malassezia* is associated with otitis and dermatitis and treatment of such disorders with antifungals often improves the conditions (Bond et al., 2020). In contrast to the situation in dogs, the association of *Malassezia* with skin disorders in humans primarily relies on clinical association studies, while a causative relationship remains a matter of debate and the mechanism of pathogenesis unclear. Dysbiosis with a higher fungal diversity and shifts in the relative abundance of certain *Malassezia* species have been implicated in AD. A large metagenomic study has reported an increase in the relative abundance of *M. dermatis* and *M. sympodialis* and a reduction of *M. globosa* on the skin of AD patients (Chng et al., 2016). Lack of consensus with other studies on the skin mycobiota in AD (Jo et al., 2017) may at least in part be due to sampling biases and discrepancies between culture- and sequence-based methods. In addition to the reported shifts in the species distribution between normal and diseased skin, intraspecies variations (Wu et al., 2015), which are known to alter phenotype and function in other fungal species (Ropars et al., 2018), may further complicate the situation. The pathogenicity of *Malassezia* spp. may also be modulated by mycoviruses that were recently identified in some isolates (Clancey et al., 2019; Park et al., 2019). Moreover, the microenvironment of the diseased skin, which is characterized by barrier disruption, lipid deficiency and elevated pH in case of AD (Weidinger and Novak, 2016), can modulate the metabolism and thereby the functional properties of the fungus (Chng et al., 2016). Inter-kingdom communications within the skin microbiota may also influence the capacity of *Malassezia* in promoting (or possibly preventing) skin disorders (Li et al., 2017). Finally, host factors such as genetics, immune status or comorbidities, can also influence the skin mycobiome composition and pathogenic potential (Jo et al., 2017) (Figure 1).

To understand the role of *Malassezia* in the development and severity of skin diseases it is important to understand the mechanisms of skin-fungus interactions in the (normal) mammalian skin.

## Host Response to the Skin Commensal Yeast *Malassezia*

Constant exposure of the skin to commensal microbes results in a continuous activation of the cutaneous immune system. Active immunosurveillance of the microbiota is critical to prevent dysbiosis, microbial overgrowth and translocation across epithelial barriers as evidenced by the frequent occurrence of opportunistic infections in immunodeficient individuals (Pellicciotta et al., 2019).

*Malassezia*-host interactions are mediated via direct contacts as well as indirectly via secreted factors (Velegraki et al., 2015) and extracellular vesicles released from the fungus, which may assist the delivery of soluble mediators to host cells (Johansson et al., 2018; Zhang et al., 2019; Vallhov et al., 2020). These interactions have been studied primarily *in vitro* with cultured cells (Sparber and LeibundGut-Landmann, 2017). Reconstructed human epidermis and *ex vivo* skin models, which reflect the complexity of the skin more closely, have also been developed to study the cutaneous antifungal response (Corzo-Leon et al., 2019; Pedrosa et al., 2019). More recently, a murine model has become available that provides insights into the host response against *Malassezia in vivo*. In contrast to *in vitro* systems, it allows to study the contribution of circulating immune cells, which are not normally resident in the skin, to the cutaneous host response over prolonged periods of time (Sparber et al., 2019).

Toll-like receptors (TLRs) and C-type lectin receptors (CLRs) recognize carbohydrates in the fungal cell wall (Plato et al., 2015). Although the cell wall of *Malassezia* shows some differences to that of other fungal genera and is covered by a lipid-rich outer layer (Mittag, 1995; Kruppa et al., 2009; Stalhberger et al., 2014), CLRs are thought to play a prominent role in *Malassezia* sensing. Dectin-2 and Mincle have both been shown to bind *Malassezia* cell wall constituents resulting in *Malassezia* phagocytosis and cytokine production (Ishikawa et al., 2013; Haider et al., 2019). However, *in vivo* the two receptors do not appear to be essential for antifungal defense because knockout mice lacking either of the two receptors do not manifest an impaired immune response in the skin (Sparber et al., 2019). Functional redundancy between the receptors and/or with other CLRs may provide a likely explanation for reconciling the involvement of the downstream signaling adaptor Card9 in the cutaneous response to *Malassezia* (Sparber et al., 2019). The requirement of MyD88 for the antifungal response (Sparber et al., 2019) may be explained by the involvement of TLR2, which is also activated by *Malassezia* (Baroni et al., 2006) (Figure 1).

Card9-mediated signaling couples innate fungal recognition to the adaptive immune system, and importantly, it drives polarization of CD4^+^ T cells into IL-17A-secreting effector cells (LeibundGut-Landmann et al., 2007). While this was first observed with *Candida albicans* in humans and mice (LeibundGut-Landmann et al., 2007; Glocker et al., 2009), it also applies to *Malassezia*: *Malassezia*-specific T helper cells belong preferentially to the Th17 subset in both host species and *Malassezia*-specific Th17 response is abolished in Card9-deficient mice (Sparber et al., 2019).

αβ T cells are not the only IL-17A-producing cell type. In the skin, γδ T cells and innate lymphoid cells (ILCs) constitute prominent additional sources of IL-17A and respond swiftly to cytokine stimulation in an antigen-independent manner (Cua and Tato, 2010). Indeed, both γδ T cells and ILCs secrete IL-17A in the skin of *Malassezia*-colonized mice (Sparber et al., 2019) (Figure 1). Whether the activation of γδ T cells and ILCs is a consequence of the experimental infection and/or whether the innate cells contribute to long-term immunosurveillance of *Malassezia* commensalism, remains to be determined. Intriguingly, the Card9 pathway does not seem to be required for induction of innate IL-17A by *Malassezia* (Sparber et al., 2019). This is reminiscent of what has been reported for innate IL-17A induction in a murine model of oropharyngeal candidiasis (Bishu et al., 2014), although the underlying cause and the Card9-independent regulatory mechanisms are unclear so far.

Complementary to classical pattern recognition receptors such as CLRs, other classes of receptors are also implicated in the recognition of *Malassezia* in the skin. They are particularly relevant in keratinocytes, which lack Syk/Card9-coupled CLRs. Examples of keratinocyte-fungal interactions are provided by studies on *C. albicans*, which implicated receptors like E-cadherin, EphA2, or EGFR in the antifungal response (Phan et al., 2007; Swidergall et al., 2018; Ho et al., 2019). In case of *Malassezia*, the aryl hydrocarbon receptor (AhR), a ligand-dependent transcription factor, has gained attention because *Malassezia*-derived metabolites, in particular indoles, can trigger AhR signaling. Indole production was first reported for *M. furfur* (Gaitanis et al., 2008), but other species of *Malassezia* may also produce AhR ligands (Magiatis et al., 2013; Buommino et al., 2018) (Figure 1). The activation of AhR signaling can modulate skin homeostasis in manifold ways, including oxidation, epidermal barrier function, melanogenesis, and innate immunity (Furue et al., 2014). Importantly, AhR is implicated in type 17 immunity by promoting Th17 differentiation (Quintana et al., 2008; Veldhoen et al., 2008) and stimulating the production of IL-17A and related cytokines by Th17 cells, innate lymphocytes and ILCs (Veldhoen et al., 2008; Martin et al., 2009; Qiu et al., 2012). Whether and how AhR signaling modulates skin immunity and in particular the type 17 response to *Malassezia* remains an open question.

Consistently with the host-protective role of the IL-23/IL-17 immune axis in barrier tissues, IL-17 cytokines (including IL-17A and IL-17F, and possibly the epithelial cell-derived family member IL-17C, which is functionally related to IL-17A and IL-17F) prevents *Malassezia* overgrowth on the murine skin (Sparber et al., 2019). This is reminiscent of the well-known activity of IL-17 against other fungi, in particular *C. albicans*, on the skin and mucosal surfaces (Sparber and LeibundGut-Landmann, 2019). In contrast to the prominent role of IL-17 in protection from mucocutaneous candidiasis, the mechanisms of immunosurveillance of *Malassezia* in humans are likely more complex. No case has been reported to date where a genetic defect in the IL-17 pathway or an upstream signaling element manifests in detectable *Malassezia* overgrowth or development of *Malassezia*-associated skin disorders. Of note, an increased incidence of seborrheic dermatitis was reported as an adverse event accompanying administration of the anti-IL-17A antibody Ixekuzumab (Saeki et al., 2017), and a genetic variant of *IL23R* was found associated with a decrease in dandruff (Ehm et al., 2017). These observations support a link between the IL-23/IL-17 immune axis and *Malassezia* in humans. More data are needed to confirm these findings. Moreover, additional immune mechanisms involved in the control of *Malassezia* commensalism await to be discovered.

## *Malassezia*-Induced Immunity and Immunopathology in the Skin

While type 17 immunity is primarily host-beneficial and supports a protective state by controlling commensal fungi in barrier tissues, IL-17 can also bear pathological potential if dysregulated. The IL-17 pathway is a strong driver of inflammation in several immune-mediated diseases, the prime example of which is psoriasis (McGeachy et al., 2019). Mechanistically, IL-17 signaling induces not only the production of antimicrobial peptides and tissue repair molecules, but also chemokines that attract inflammatory myeloid cells to the tissue and it promotes hyperproliferation of keratinocytes. The causative role of IL-17 in psoriasis is demonstrated by the therapeutic efficacy of neutralizing antibodies targeting IL-17 family cytokines and receptors (Hawkes et al., 2017). Although the initial trigger of the pathological IL-17 response in psoriasis is unknown, (skin) fungal commensals are likely involved due to their prominent IL-17-inducing capacity.

IL-17 has also been linked to AD (Koga et al., 2008), although the causative relationship between the cytokine and disease pathogenesis is less clear. It appears to be of particular importance in subtypes of the disease (Leonardi et al., 2015; Noda et al., 2015; Esaki et al., 2016). *Staphylococcus aureus* colonization of the skin is among the most well-known hallmarks of AD (Kong et al., 2012), and the induction of IL-17 in response to epicutaneous *S. aureus* critically mediates skin inflammation in an experimental setting (Liu et al., 2017; Nakagawa et al., 2017). Likewise, *Malassezia* promotes skin inflammation under AD-like conditions via the IL-23/IL-17 immune axis (Sparber et al., 2019). Support for the contribution of *Malassezia*-induced IL-17 to disease pathogenesis is further provided by the observation that *Malassezia*-specific Th17 cells are enriched in AD patients (Balaji et al., 2011; Sparber et al., 2019). Beyond AD, a link between the IL-23/IL-17 immune axis and *Malassezia*-associated disorders has also been proposed in seborrheic dermatitis (Wikramanayake et al., 2018) and dandruff (Ehm et al., 2017). Besides the prominent IL-17 profile, *Malassezia*-responsive T cells in humans also produce IFN-γ (Balaji et al., 2011; Bacher et al., 2019; Sparber et al., 2019), whereby the reason underlying the discrepancy between the two host species remains unclear.

The association of *Malassezia* with AD was first based on the observation that AD patients are often sensitized to *Malassezia* with Th2 cells and IgE that are directed against the fungus (Scalabrin et al., 1999; Zargari et al., 2001; Johansson et al., 2002; Balaji et al., 2011). The *Malassezia*-specific IgE titers were found to correlate with the severity of AD (Zhang et al., 2011; Glatz et al., 2015), albeit their pathogenic role in AD is not well-understood. An increase in *Malassezia*-specific serum IgE was also documented in canine AD (Khantavee et al., 2019) (Figure 1).

Important questions arise as to how *Malassezia*-specific Th2 cells are primed and whether they are a cause or a consequence of the allergic response in the atopic skin environment. It also remains unclear how the mixed Th2/Th17 response develops in allergic individuals and how the Th cell subsets are related to each other. Possible non-exclusive scenarios comprise that they may result from differential polarization or selective outgrowth of specific subsets. Clonotypic analysis of *C. albicans*-specific T cell subsets revealed intraclonal functional heterogeneity in support of a scenario of preferential outgrowth of individual subsets (Becattini et al., 2015). Mixed Th2/Th17 populations may also arise from T cell plasticity with intermediate T cells co-producing IL-4 and IL-17A and co-expressing GATA3 and RORγt as they have been observed in the blood and airways of asthmatic patients (Cosmi et al., 2010; Wang et al., 2010; Irvin et al., 2014) and in the skin of atopic individuals (Roesner et al., 2015).

Regarding the mechanism how *Malassezia*-specific T cells promote the allergic inflammation, it was speculated that cross-reactivity may be involved and thereby add an autoimmune component to the pathogenicity of AD. Among the *Malassezia*-derived antigens that have been identified are some that are phylogenetically highly conserved in mammals, such as thioredoxin and manganese-dependent superoxide dismutase (Schmid-Grendelmeier et al., 2005; Glaser et al., 2006; Balaji et al., 2011). Alternatively, *Malassezia*-specific T cells may also mediate immunopathology via cross-reactivity with other microbes as recently reported for *C. albicans*-specific T cells (Bacher et al., 2019). The emerging concept of cytokine-mediated and T cell receptor-independent bystander activation of T cells may represent another putative mechanism (Lee et al., 2019).

Given the pathogenic potential of the *Malassezia*-specific immune response, tight regulation is required to maintain commensalism. Induction of immune tolerance to skin commensal microbes has been investigated in case of *Staphylococcus epidermidis*. Antigen-specific regulatory T cells (Tregs) that accumulate during a critical developmental window in neonatal life were found important to balance the antimicrobial Th17 effector cells directed against the commensal (Scharschmidt et al., 2015). In a model of fungal commensalism with *C. albicans* however, Tregs and regulatory cytokines such as IL-10 appeared not essential for immune homeostasis and stable persistence of the fungus in the oral mucosa (Kirchner et al., 2019). Whether and how immune regulation contributes to immunosurveillance and prevents immunopathology in case of *Malassezia* remains to be determined.

The pathogenic effects of *Malassezia* in case of AD and other inflammatory skin disorder may also be mediated by T cell-independent mechanisms. *Malassezia* may promote disease by triggering the production of pro-inflammatory mediators in keratinocytes and tissue-resident immune cells in the atopic skin that are more accessible to the fungus, owing to barrier defects. *Malassezia* itself produces lipases and phospholipases that generate free fatty acids, which can damage the integrity of the skin and thereby might contribute to irritation and inflammation (Dawson, 2007; Saunders et al., 2012). *Malassezia* proteases may also interfere with cutaneous wound healing by degrading extracellular matrix components (Poh et al., 2020). While these various *Malassezia*-derived components have been studied *in vitro*, their relevance for promoting inflammation in the skin remains unclear. Complementary to the above described scenarios, *Malassezia* may also influence disease by modulating the virulence of other skin microbes such as *S. aureus* via inter-kingdom interactions (Li et al., 2017). It was postulated that such interactions could possibly be host beneficial rather than disease promoting by attenuating bacterial biofilm formation (Li et al., 2017), or by mechanisms such as those reported for *Staphylococcus* interspecies interactions (Nakatsuji et al., 2017).

## *Malassezia* and Disease Beyond the Skin

Aside from its abundance on the skin, *Malassezia* has also been detected in extracutaneous sites. In particular, *Malassezia* sequences have been identified in the gastrointestinal tract in association with inflammatory bowel disease (IBD). Fungal dysbiosis in IBD is characterized with an enrichment of fungal taxa belonging to the Basidiomycota phylum, and this effect was balanced by an equivalent decrease in taxa belonging to the Ascomycota phylum (Sokol et al., 2017). More recently, in an ITS1 sequencing analysis of intestinal washings, *Malassezia* was associated with a subgroup of Crohn's disease patients carrying the *CARD9* risk allele (Limon et al., 2019). Among the *Malassezia* sequences, *M. restricta* was most abundant. Relevance for the role of *M. restricta* in gut inflammation was provided by the observation that gastrointestinal delivery of the fungus aggravates the outcome of DSS-induced colitis in mice in a Card9-dependent manner (Limon et al., 2019). This phenotype was further linked to the induction of Th17 cells by *M. restricta* in the colonic lamina propria (Limon et al., 2019), whereby IL-17 is involved in the pathogenesis of disease in this model (Ito et al., 2008). It remains unclear whether the detection of *Malassezia* by sequencing approaches reflects transient relocation or true colonization of the gut by the fungus. In addition, it is unknown whether *Malassezia*-specific Th17 cells are involved in the pathogenesis of Crohn's disease, and if so, whether these cells get primed locally in response to fungal dysbiosis, or whether they translocate from the skin.

That Th17 cells directed against commensal fungi act at distal sites from where they have been induced has also been observed in other contexts with either host-protective or pathogenic consequences. In gut colonization experiments, *C. albicans*-induced Th17 cells protect from systemic candidiasis in a T cell- and IL-17-dependent manner (Shao et al., 2019). The protective effect extends to other extracellular pathogens, such as *S. aureus* (Shao et al., 2019). These effects are complemented by harmful consequences such as increased susceptibility to airway inflammation and exacerbation of asthma-like symptoms (Noverr et al., 2004; Shao et al., 2019). Besides these examples from experimental models of *C. albicans* colonization in mice, *C. albicans*-specific Th17 cells in human were also shown to cross-react with *A. fumigatus* and to expand in patients with *A. fumigatus*-driven lung pathology, suggesting an involvement in allergic asthma (Bacher et al., 2019). Examples of a skin-lung axis have been provided by bacterial skin commensals (Fyhrquist et al., 2014). It is tempting to speculate that Th17 cells primed against *Malassezia* in the skin may act in a similar way to promote the progression from AD to allergic asthma and rhinitis and thereby contribute to the atopic march.

Beyond its impact on the pathogenesis of inflammatory diseases, *Malassezia* was recently implicated in carcinogenesis. Among the microbes infiltrating pancreatic ductal adeno-carcinomas (PDA) tumors, *Malassezia* was markedly enriched (Aykut et al., 2019). In a murine model of PDA, *Malassezia* exerted a tumor-promoting effect via a mechanism involving mannose-binding lectin, a CLR and activator of complement and implicated in antifungal innate immunity (Aykut et al., 2019). *Malassezia* is likely a ligand of MBL, although direct binding has not been demonstrated. Whether fungal dysbiosis is cause or consequence of oncogenesis is not fully clear. The link between *Malassezia* and carcinogenesis may be more general, as fungal dysbiosis was also observed in colitis-associated cancer with an enrichment of *Malassezia* in the colonic mucosa associated fungal microbiota (Richard et al., 2018).

## Concluding Remarks

Recent advances in the field have shed light on the immune response to *Malassezia* in the skin and on the divergent consequences that the antifungal response can have on the host. IL-17-dependent immunity against *Malassezia* contributes to the maintenance of fungal commensalism and skin homeostasis. The same pathway however can also have host-adverse effects in predisposed individuals, and this effect is likely not limited to the skin but also affects extracutaneous sites. The factors determining these context-dependent outcomes remain largely unclear. Moreover, it remains unknown to what extent the effects of the antifungal immunity on disease pathogenesis is shared or distinct between different *Malassezia*-associated disorders. Beyond immunological pathways, non-immunological factors such as neurotransmitters and possibly hormones affect the antifungal response, and this is likely also the case with *Malassezia*. Consistently, neuroendocrine changes are known to impact the course and severity of *Malassezia*-associated skin disorders. Future research will inform about the potential of therapeutically targeting fungal communities or the antifungal response for improving patient outcome.

## Author Contributions

FS and SL-L wrote the minireview. FR created the figure with BioRender.com. All authors reviewed the manuscript.

## Conflict of Interest

The authors declare that the research was conducted in the absence of any commercial or financial relationships that could be construed as a potential conflict of interest.
